# Bromelain-functionalized omega-3 nanocarriers for targeted icariin delivery: a multifunctional shield for cardiac repair in doxorubicin-induced injury

**DOI:** 10.1007/s10856-025-06915-1

**Published:** 2025-12-06

**Authors:** Nermeen H. Kamal, Lamia A. Heikal, Maged W. Helmy, Ossama Y. Abdallah

**Affiliations:** 1 Division of Pharmaceutical Sciences, Department of Pharmaceutics, College of Pharmacy, Arab Academy for Science, Technology and Maritime Transport, Alexandria, Egypt; 2 Department of Pharmaceutics, Faculty of Pharmacy, Alexandria University, Alexandria, Egypt; 3 Department of Pharmacology and Toxicology, Faculty of Pharmacy, Damanhour University, Damanhour, Egypt

## Abstract

The major loss of cardiomyocytes caused by several cardiovascular diseases as myocardial infarction, increases the incidence of heart failure. Thus, there is an urgent need to develop novel therapies that can enhance angiogenesis and decrease the rate of cardiac cell apoptosis. The use of nanoparticles that can passively target infarcted myocardium can be appealing. Icariin is a natural product with various cardioprotective properties that can be encapsulated within nanostructured lipid carriers (NLCs) to enhance its therapeutic effect. Incorporating bioactive excipients such as omega-3 oils and bromelain into the NLC formulation can potentiate its cardioreparative effects. Therefore, the use of such nanoformulation can be an attractive option to minimize cardiac damage. Optimized bromelain-coated and uncoated NLCs loaded with icariin were successfully developed, demonstrating efficient bromelain surface modification, high drug entrapment, and a favorable release profile. In vitro experiments using doxorubicin (DOX)-treated H9c2 cardiomyocytes highlighted the superior cellular uptake of NLCs compared to the free solution. Notably, pretreatment with optimized bromelain-coated and uncoated icariin-loaded NLCs significantly improved cell viability and reduced apoptotic rates, indicating their potential role in cardioprotection. The therapeutic effect of NLCs was markedly enhanced relative to free icariin, demonstrating the added value of nano-formulation. Combination index (CI) analysis using Compusyn further verified the synergistic interaction between nano-formulated icariin and bioactive excipients, enabling improved therapeutic outcomes with lower effective doses. These findings highlight the potential of NLC-based delivery systems in counteracting doxorubicin-induced cardiotoxicity, supporting further preclinical studies for clinical translation.

**Graphical Abstract:**

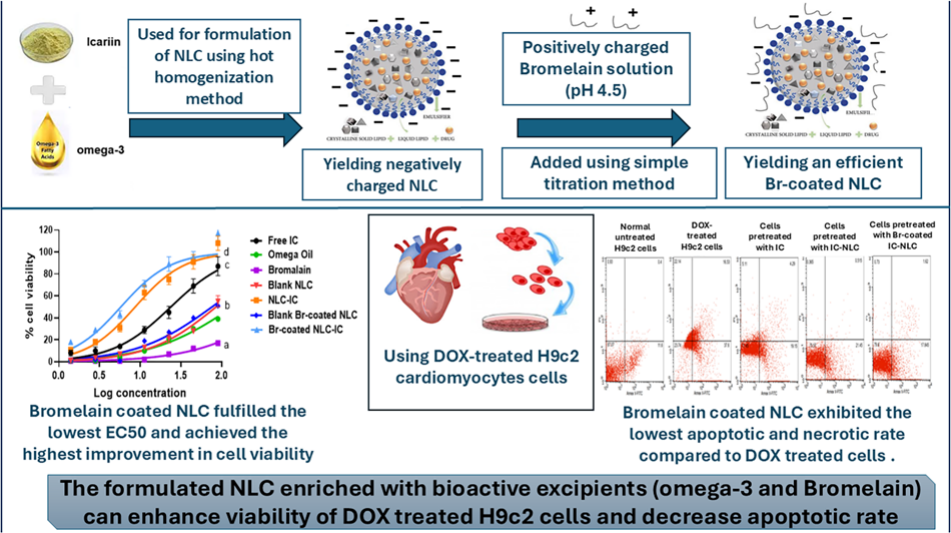

## Introduction

Heart failure (HF) is a consequence of massive loss of cardiomyocytes caused by various cardiovascular diseases, particularly myocardial infarction (MI). Despite the successful early management of MI by percutaneous coronary intervention technique, the incidence of HF remains common [1]. The prognosis of HF has been considered worse than many types of cancers, with a survival rate of 50% over 5 years. The current therapies managed to reduce the mortality rate immediately after the incidence of MI; however, they did not address the underlying loss of cardiomyocytes and vasculature. Thus, they failed to prevent or delay the progression of HF. Notably, HF is a leading cause of hospitalization, with extensive healthcare costs that are expected to exceed $70 billion by 2030 in the United States [2].

The need to develop novel therapies for HF post-MI that can address the loss of cardiomyocytes is impelling. Acute MI can kill up to 1 billion cardiomyocytes from the left ventricle, representing around 25% of these cells [3]. Moreover, the chronic myocardial disease and ischemia may extend the death of cardiac cells over prolonged periods of time. Consequently, this loss of cardiomyocytes negatively deteriorates the contractile tissue of the myocardium. The concept of cardiac regeneration can be achieved by two strategies to restore the contractile ability of the heart. First, the use of diverse cell-based therapies can be administered to the heart. However, this strategy has faced multiple challenges, such as immunosuppressing the recipient to accept injected cells, the incidence of arrhythmias, and the poor survival rate of administered cells. Alternatively, the second strategy aimed to stimulate the endogenous capacity of cardiomyocytes to proliferate through the administration of different genes, RNAs, or drugs [4].

The use of nanotechnology-based drug delivery to deliver cardioprotective agents to the infarcted myocardium can be appealing. Icariin “IC” is a Chinese phytomedicine with various cardioprotective properties. It was reported that IC can mitigate oxidative stress, reduce harmful inflammatory responses, promote angiogenesis, and regulate cell apoptosis [5]. IC plays a crucial role in preventing cardiomyocyte apoptosis induced by endoplasmic reticulum stress (ERS) by inhibiting proteins such as glucose-regulated protein 78 and caspase-3, which in turn could reduce reactive oxygen species (ROS) production and minimize ERS-induced cardiomyocyte damage [6]. IC had demonstrated promising potential for ventricular remodeling and fibrosis inhibition through the regulation of the TGF‐β/Smad signaling pathway. Furthermore, Sharma et al. proposed that IC counteracted isoproterenol-induced cardiotoxicity by increasing myocardial cyclic guanosine monophosphate levels via phosphodiesterase-5 inhibition and reducing nuclear factor-kappa B expression [7]. Therefore, IC presents a promising therapeutic option for myocardial infarction treatment.

The infarcted heart can be achieved by either passive or active targeting after intravenous administration of formulated nanoparticles. The intravenous route of administration is considered the least invasive route compared to intramyocardial injection. Lipid-based nanocarriers are biocompatible, biodegradable, and can encapsulate both hydrophilic and hydrophobic compounds. They are characterized by the relative simplicity of their preparation, low production cost, and scaling up. Lipid nanocarriers have been extensively studied and are clinically applied in various types of cancer. Interestingly, they are also promising candidates for drug delivery to the infarcted heart [8]. The intravenous administration of lipid-based nanocarriers has been shown to successfully deliver cardioprotective drugs to the infarcted myocardium by passive targeting [9].

Solid lipid nanoparticles (SLNs) are derived from oil-in-water nano-emulsions, in which liquid oil is replaced by lipids that are solid at room temperature. The advantages of SLNs include low toxicity, avoidance of organic solvents, and good in vivo tolerance [10]. These nanocarriers can improve the bioavailability of the incorporated active pharmaceutical ingredient (API) and can protect APIs liable to degradation, thus improving their stability. However, limited drug loading and the possibility of drug expulsion during storage are considered the main disadvantages of this system. This is mainly attributed to the crystalline nature and possible polymorphic transitions of solid lipids that limit available space for drug loading.

Nanostructured lipid carriers (NLCs) were introduced as the second generation of lipid-based nanoparticles. They were developed to resolve the problems raised by SLNs [11]. The main difference between NLCs and SLNs is the inner structure. NLCs contain a certain amount of liquid lipid, usually up to 30%. The incorporation of these small amounts of oil within a solid lipid matrix can improve the loading capacity of the system in comparison to SLNs. This oil can distort the perfect crystalline nature of solid lipid, causing imperfections and providing space to incorporate more drug molecules in amorphous clusters [12].

NLCs were selected as good candidates to encapsulate IC and passively target infarcted myocardium through the enhanced permeation and retention phenomenon. This selection was recommended as these systems offer safe, non-toxic, biodegradable, and biocompatible drug delivery. Moreover, incorporating bioactive excipients that can enhance cardiac healing in these nano-formulations would be advantageous. Besides the role of these nano-systems in improving poor physical properties and sustaining the release of IC, they will aid in achieving the required therapeutic effect. The selection of NLCs was favored over polymeric and metal nanoparticles for several reasons. First, natural polymers have significant drawbacks such as rapid degradation, immunological reaction, and poor mechanical properties. Synthetic polymers, on the other hand, suffer from low biocompatibility, a lack of cell attachment, and high production cost [13, 14]. Regarding metal nanoparticles, despite their beneficial effects, more intense research is required to investigate their toxicity profile and the hazards associated with the relative accumulation of metals within cardiac tissues [15].

Omega-3 (n-3) is an essential fatty acid extracted from fish oil. Fish n-3 fatty acids include eicosapentaenoic acid (EPA) and docosahexaenoic acid (DHA). Omega-3 has demonstrated antioxidant, anti-apoptotic, and anti-inflammatory effects in cardiovascular disorders, with studies highlighting their potential in reducing oxidative stress, promoting myocardial cell differentiation, and mitigating cardiotoxicity. Given these cardioprotective benefits, omega-3 oil was selected as the bioactive liquid lipid in the formulation of NLC [16, 17].

Bromelain (Br) is a proteolytic enzyme extracted from the stem of the pineapple. This extract exhibits fibrinolytic, antiedematous, antithrombotic, and anti-inflammatory effects. Therefore, Br was recognized as a useful phytomedicine with variable applications [18]. It was reported that Br can induce disruption of thrombus, reduce blood viscosity, and inhibit platelet aggregation [19]. Moreover, Juhasz et al. reported the ability of Br to limit myocardial injury post-reperfusion. Thus, Br treatment managed to reduce infarct size and apoptosis rate [20]. Thus, Br was selected as another bioactive excipient that can be added within NLC to potentiate its therapeutic effect.

In this study, our aim was to prepare a nano-formulation whose size is smaller than 200 nm to passively target the infarcted myocardium. NLC was formulated using bioactive omega-3 as a liquid lipid and loaded with phytomedicine “Icariin” to enhance cardiac regeneration post infarction. For the first time, Br was used to coat NLC depending on electric deposition to introduce Br as a second bioactive excipient. The formulation was subjected to in vitro characterization, and its therapeutic effect was assessed using several cell culture experiments.

## Materials and methods

### Materials

Icariin (purity 98%) and Stem Bromelain (2400 gdu/g) were purchased from Baoji Guokang Biotechnology, China. Compritol 888 ATO was received as a gift from Gattefosse, Nanterre, France. Omega-3 fish oil was received as a gift from Lysi, Iceland. Poloxamer 407 was purchased from BASF, Germany. Tween 80 was purchased from Sigma-Aldrich (St Quentin-Fallavier and Yvelines, France). All organic solvents used were of analytical grade.

### Preparation of nanostructured lipid carriers (NLCs)

Blank and IC-loaded NLCs were prepared by the hot homogenization method as reported in our previous study [21] with some modifications. Compritol® represented the solid lipid, and omega-3 oil was the selected liquid lipid. The lipid phase concentration was adjusted to 3% w/v. Different solid lipid: oil ratios (90:10, 70:30, 60:40, and 50:50 w/w) were investigated as illustrated in Table 1. Briefly, the lipid phase mixture was set to melt in a thermostatically controlled water bath adjusted to a temperature of 5 °C above the melting point. A surfactant solution in deionized water was heated at the same temperature, then added to the molten lipid and stirred at 400 rpm at 75 °C for 10 min using a magnetic stirrer. The mixture was then instantly homogenized using a high-shear homogenizer at 15,000 rpm at 75 °C using a water bath for two cycles, each 5 min. The final volume of the formulation was completed to 10 mL with deionized water. The formulation was subjected to cold water to reach ambient temperature. For formula optimization, two surfactants were investigated: Poloxamer 407 and Tween 80 at different concentrations, including 0.5, 1, and 1.5% w/v. To prepare the drug-loaded NLCs, the same procedure was followed, and IC was added to the lipid phase during melting. Coumarin (C-6) was added to the lipid mixture instead of IC in cellular uptake experiments.

**Table 1:** Optimization of NLC using different solid lipid: oil ratios and different surfactants with varying concentrations per 10 mL formulation, while keeping the total amount of lipid constant at 3% w/v

Formulation code	Blank or loaded with 3 mg IC	Lipid: oil ratio			Surfactant			Concentration of SAA
		90:10	70:30	60:40	50:50	Poloxamer 407	Tween 80	
F1	Blank		√			√		0.5%
F2	Blank		√			√		1%
F3	Blank		√			√		1.5%
F4	Blank		√				√	1%
F5	IC		√				√	1%
F6	IC	√					√	1%
F7	IC			√			√	1%
F8	IC				√		√	1%

### Preparation of bromelain-coated NLCs (Br-NLCs)

For Br-NLCs, they were prepared by a simple titration method that was previously reported for chitosan coating [22, 23], and used for the first time with some modifications to obtain Br-coated NLCs. Briefly, different volumes of Br solution in phosphate buffer (pH 4.5 and pH 6) at different concentrations (0.2, 0.5, 0.7, and 2% w/v) were added dropwise to 1 mL of IC-NLCs as shown in Table 2. The mixture was kept under mild magnetic stirring (500 rpm) at room temperature for 1 h. Effective Br coating was confirmed by size and ζ-potential measurements [24].

**Table 2:** Optimization of Br-coated NLCs using different Br solution concentrations, pH, and variable volume ratios

Formulation code	pH of Bromelain solution		Concentration of Bromelain solution (%)	Volume ratio (Br solution: Formulation)
	4.5	6		
S1	√		0.2	0.5:10
S2	√		0.2	1:10
S3	√		0.2	2:10
S4	√		0.5	0.5:10
S5	√		0.5	1:10
S6	√		0.5	2:10
S7	√		0.7	0.5:10
S8	√		0.7	1:10
S9	√		0.7	2:10
S10		√	0.2	0.5:10
S11		√	0.2	1:10
S12		√	0.2	2:10
S13		√	0.5	0.5:10
S14		√	0.5	1:10
S15		√	0.5%	2:10
S16		√	2%	1:10
S17		√	2%	1.5:10
S18		√	2%	2:10

### In vitro characterization of NLCs

#### Colloidal properties of uncoated NLCs and Br-coated NLCs

The particle size, polydispersity index, and zeta potential of IC-NLCs and Br-coated NLCs were determined using Malvern Zeta Sizer by dynamic light scattering at 25 °C. Formulations were diluted 1:100 v/v with deionized water and sonicated for 5 min prior to measurements. All measurements were done in triplicate at ×30 magnification power.

#### Determination of drug entrapment efficiency (EE%)

The EE% was determined using an indirect method previously reported and validated with some modifications [25]. The IC-NLCs were separated using an ultrafiltration technique to determine the concentration of free unentrapped drug in the filtrate. In brief, a certain volume (0.2 mL) of NLC was placed within the upper chamber of an ultracentrifugal concentrator (SartoriusTM Vivaspin 6TM, MWCO 100,000) and diluted with deionized water for 10 folds. The Vivaspin tubes were centrifuged for 1 h at 6000 rpm at room temperature. An aliquot of filtrate was investigated using UV-Vis spectrophotometer (T80 series of UV-Visible Spectrophotometers (pg instruments, UK)), at 270 nm to determine the concentration of the unentrapped drug. The EE% of Br-coated IC-NLCs was also carried out to investigate the effect of Br coating on the entrapment of IC. Samples were measured in triplicate and represented as mean value ± SD. The concentration of free drug was calculated as the difference between the total amount of drug and the amount of unentrapped free drug according to Equation (1):

$${{EE}} \% =\frac{W\left({{{added}}\; {{drug}}}\right)-W({{{free}}\; {{drug}}})}{W({{{added}}\; {{drug}}})}* 100$$

Where *W* (added drug): the total amount of drug added to the nanocarriers, and *W* (free drug): the unentrapped drug content in supernatant.

#### Morphological examination of NLC and Br-NLC using transmission electron microscope (TEM)

The morphology of the prepared IC-NLCs and Br-IC-NLCs was examined by TEM. The nano-dispersions were properly diluted (1:200 v/v) with filtered distilled deionized water. Formulations were dropped onto copper grids, stained with 2% w/v aqueous solution of uranyl acetate, and left for a few minutes to dry out before examination.

#### Determination of Br coating efficiency using Bradford reagent

The Br coating efficiency was determined using a Vivaspin ultrafiltration method and Bradford assay for protein quantification. A known volume of Br-NLC (2 mL) was placed within the upper chamber of the ultracentrifugal concentrator (SartoriusTM Vivaspin 6TM, MWCO 100,000). The Vivaspin tubes were centrifuged for 1 h at 6000 rpm at room temperature. An aliquot of filtrate (0.1 mL) was then mixed with 3 mL of Bradford reagent. The mixture was incubated for 5 min, and the absorbance of the samples was measured using a UV-Vis spectrophotometer at 595 nm. The concentration of protein was determined using a calibration curve constructed using serial dilutions of albumin (0.1–1.4 mg/mL). The concentration of free bromelain was calculated as the difference between the total amount of protein and the amount of unentrapped free Br, as shown in equation 1 (section “Determination of drug entrapment efficiency (EE%)”).

#### Fourier transform infrared spectroscopy (FTIR) spectrum

FTIR analysis was used to confirm the successful surface modification of unloaded NLC and IC-NLC with Br and to investigate molecular interactions. The IR spectra were recorded on an Agilent Technologies Carry 630 FTIR. Spectral scanning was done in the range between 4000 and 500 cm^−1^. FTIR chart was recorded for the following: IC, Br, physical mixture of Br with lyophilized uncoated NLC, uncoated /Br-coated IC-NLC, and Br-coated blank NLC.

#### Differential scanning colorimetry (DSC) study

The curves of IC, Br, lyophilized Br-coated blank NLC, and Br-coated IC-NLC were obtained by placing in an aluminum crucible and heating at a rate of 10 °C/min from 0 °C to 400 °C under a nitrogen atmosphere with flow rate of 150 mL/ min using differential scanning calorimetric instrument (DSC-4000, Perkin Elmer, USA).

#### X-ray diffraction (XRD) of optimized Br-coated NLC loaded with IC

XRD measurements of crude IC, Br, and lyophilized Br-coated NLC loaded with IC were conducted on a MeaSrv (D2–208219) diffractometer with an X-ray source of Cu Kα radiation (*λ* = 1.54184 Å) and Lynxeye as a detector. The apparatus operated with a voltage of 30 kV and a current of 10 mA. Data was collected in 17 min using a 2 Theta range from 0.998 to 100° and a step size of 0.01° with 30 kV and 10 mA. The scanning scope of 2*θ* was 5–50°, and the scanning rate was 5°/min.

#### In vitro release study

##### The solubility study of IC in different release media

The solubility of IC was evaluated in various release media for the selection of a suitable medium to study its in vitro release from different prepared formulations. The tested media included: phosphate-buffered saline (PBS, pH 7.4), 0.5% and 1% w/v Tween 80 in PBS, as well as PBS containing 15% v/v and 30% v/v methanol. An excess amount of IC (20 mg) was added to 5 mL of each medium in separate vials. These vials were placed in a shaking water bath (Model: LSB-030S, DAIHAN LABTECH CO., LTD, Republic of Korea) at 37 °C and agitated at 100 rpm for 24 h. Afterward, the samples were maintained at the same temperature without shaking for an additional 24 h to reach equilibrium. The supernatant was then collected, appropriately diluted, and analyzed using a UV-Vis spectrophotometer at 270 nm.

##### In vitro release profile of IC from IC-NLC and Br-coated IC-NLC

The in vitro release study was carried out using the dialysis bag method (MWCO 8000–14000 Da) for IC-loaded NLC and Br-coated NLC loaded with IC. A 30% v/v methanol in phosphate buffer saline medium (PBS, pH 7.4) was selected to achieve the required sink conditions according to the solubility study.

Briefly, a known volume of IC solution in 30% v/v methanol in PBS, IC suspension in PBS, IC-NLC, and Br-IC-NLC equivalent to 1 mg IC were placed in a dialysis bag and placed in 20 mL release medium in a shaking water bath at 37 °C and 100 rpm to ensure sink condition. The release medium (1 mL) was withdrawn at 0.25, 0.5, 1, 2, 3, 4, 5, 6, and 24 h and replaced with fresh release medium to maintain the sink conditions. The withdrawn aliquots were examined using a UV-Vis spectrophotometer [26], and measurements were done at 270 nm. The analytical method was validated in terms of linearity, sensitivity, and limit of detection. The percentage cumulative drug released was calculated and plotted against time. The mechanism of IC release from the uncoated and Br-coated NLC was analyzed using the Excel add-in DD solver by fitting to different release kinetics models. The in vitro release profile was fitted to zero-order, first-order, Higuchi, Korsmeyer-Peppas, Hixon-Crowell, and Weibull models, and the best fit was determined based on the calculated highest correlation coefficient (*R*^2^) and lowest Akaike information criterion (AIC).

#### Stability study

The optimum uncoated NLC formulation (*F4*) and the coated one (*S6*) were stored for 3 months in tightly closed containers in a refrigerator (4 ± 2 °C). The stored nanocarriers were assessed monthly for changes in their macroscopic appearance, PS, PDI, and ZP.

### Cell culture

Different cell culture experiments were conducted on H9c2 primary cell line of rat cardiomyoblasts (Nawah Scientific, Egypt) that was cultured in Dulbecco’s modified Eagle’s medium (DMEM) containing 10% fetal bovine serum, 1% or 100 U/mL penicillin/streptomycin at 37 °C in a humidified 5% CO_2_ atmosphere.

#### Cell viability assay

The effect of bioactive excipients (omega-3 and Br), IC solution, and IC-loaded formulations on H9c2 cell viability was evaluated using the MTT test. H9c2 cells were cultured in 96-well plates at a seeding density of 5 × 10^3^ cells/well. The H9c2 cells were treated with doxorubicin (Dox) to reduce their viability and mimic cardiac toxicity conditions. The effect of pretreating Dox-treated cells with the different formulations on enhancing their viability was investigated. Cells were pretreated with serial dilutions of free IC in DMSO, omega-3 diluted in DMSO, Br solution in buffer, blank/IC-loaded NLC prepared using omega-3 as liquid lipid, and Br-coated blank/IC-loaded NLC formulations. The concentrations used for the different formulations were 1.4, 2.8, 5.625, 11.25, 22.5,45 and 90 µM. The formulations were applied and incubated for 3 h; subsequently, 1 µM DOX was added. Finally, the cells were incubated for 24 h.

At the end of the treatment, the medium was replaced by the water-soluble dye 3-(4,5-dimethylthiazol-2-yl)-2,5-diphenyltetrazolium bromide (MTT) solution (5 mg/mL) and incubated for 4 h. After the incubation, 100 µL of DMSO was added to solubilize the formed formazan crystals. Absorbance was measured using an automated microplate reader (BioTek Instruments, VT, USA) at 570 nm to calculate the percentage of cell viability with respect to control untreated cells and control doxorubicin cells, respectively [27].

For the analysis of the synergistic effect of different bioactive excipients (omega-3 and Br) in addition to IC on the enhancement of DOX-treated H9c2 cells’ viability, CompuSyn software was employed [28]. Combination index (CI) was calculated according to equation (2), where CI < 1, = 1, or > 1 represents synergistic protective effect, additive protective effect, or antagonism, respectively [29].

*Equation 2: Combination index*

$${{CI}}=E\left({ca}\right)* E\left({da}\right)+E\left({cb}\right)* E({db})$$

Where CI = combination index, *E*(*ca*) = effect for drug *a* in combination, *E*(*cb*) = effect for drug *b* in combination, *E*(*da*) = effect of drug *a* alone, and *E*(*db*) = effect of drug *b* alone.

Also, dose reduction indices (DRI) were calculated, indicating the fold-decrease in the dose of the IC, omega-3, and Br when applied separately relative to their dose in combination within NLC to achieve the same protective effect [30]. A DRI value greater than 1 indicates effective dose reduction of drugs in a mixture compared to their individual.

#### Cellular uptake

Cellular uptake of uncoated NLC and Br-coated NLC loaded with the fluorescent dye, coumarin-6, instead of IC (0.4 µg/mL) in addition to free coumarin-6 (C6) solution in DMSO (0.4 µg/mL) was assessed using DOX (1 µM) treated H9c2 cells. H9c2 cells were cultured at a seeding density of 5 × 10^5^ cells/well in a 6-well plate. After 24 h incubation, C6-loaded formulations and free C6 were co-added with DOX (1 µM) to reach a final concentration of 100 ng/mL of C6 in cells. The formulations and free C6 were incubated with the cells for 4 h. Cells were washed several times with PBS, then fixed with 4% v/v paraformaldehyde in PBS solution at room temperature and left for 15 min. A confocal laser scanning microscope (CLSM) (LeicaR Microsystems Inc. Model DMi8, Metzler, Germany) was used to measure the fluorescence intensity of C6 fluorescent dye at 355 nm to assess the cellular uptake. All experiments were done in protected conditions from direct light. Nuclei were stained with Höechst 33342. The blue fluorescence intensity of Höechst 33342 was measured at 358/416 nm, and the green fluorescence intensity of C6 in images was quantified using ImageJ 1.52a software obtained from the National Institutes of Health, USA, to determine fluorescence intensity [31].

#### Apoptosis rate (Annexin V FITC/Propidium iodide assay)

Apoptosis was evaluated by Annexin V assay by flow cytometry (BD FACSCalibur™ flow cytometer, San Jose, USA). Briefly, cells were cultured in 6-well plates at a seeding density of 5 × 10^5^ cells/well. After 24 h incubation at 37 °C, cells were pretreated with 15.4 µM IC (EC_50_) solution or an equivalent amount of Br-coated and uncoated blank/IC-loaded NLC prepared with omega-3 for 3 h, then DOX (1 µM) was added, and cells were incubated for 24 h. Subsequently, cells were then trypsinized, collected after centrifugation at 2000 rpm, and stained with Annexin V FITC and ropidium iodide (PI) according to the manufacturer’s protocol. Normal untreated H9c2 cells were considered the negative control, and DOX-treated cells were identified as the positive control. The cells were double-stained with Annexin V/PI and analyzed by flow cytometry. Annexin V has affinity for phosphatidylserine and indicates early apoptosis. While PI is a nucleic acid dye that cannot penetrate an intact cell membrane, thus, it thus indicates late stages of apoptosis. Untreated normal H9c2 cells and DOX-treated cells were also subjected to the previously mentioned methodology. Analysis of apoptotic cells was done by 20,000 cells gating by flow cytometry. The experiment was done in triplicate (*n* = 3), and representative images were provided.

### Statistical analysis

All data were expressed as means ± standard deviations (SD). Comparison of data groups was performed by one-way ANOVA followed by post-hoc comparisons (Tukey test) using GraphPad Prism 8.4.3 (GraphPad software, Boston). Significant differences were considered at *p* ≤ 0.05.

## Results

### Preparation and optimization of blank and IC-loaded NLCs

During optimization of the NLC, Poloxamer 407 and Tween 80 were screened as stabilizers using various concentrations: 0.5, 1, and 1.5% w/v. F1–F3 were blank NLCs prepared using poloxamer 407 as a surfactant using different concentrations, 0.5,1 and 1.5% w/v, respectively. F3, which was prepared using the highest concentration of poloxamer 407, fulfilled the smallest particle size of 153.9 ± 15.4 nm and PDI value of 0.3 ± 0.1 (*P* < 0.05) as shown in Table 3. F4, which was prepared using 1% w/v Tween 80, exhibited a nano-formulation within an acceptable size of 160.2 ± 12.3 nm with a PDI value of 0.2 ± 0.1.

**Table 3:** Colloidal properties of NLCs during formula optimization

Formulation code	Particle size (PS)	Polydispersity index (PDI)	Zeta potential
F1	603.8 ± 20.1	0.5 ± 0.04	−30.2 ± 15.3
F2	237.6 ± 10.2	0.3 ± 0.1	−34.7 ± 20.1
F3	153.9 ± 15.4	0.3 ± 0.1	−31.5 ± 10.2
F4	160.2 ± 12.3	0.2 ± 0.1	−30.1 ± 12.4
F5^a^	173.9 ± 8.9	0.2 ± 0.05	−30.2 ± 1.2
F6	234.1 ± 11.4	0.3 ± 0.03	−34.8 ± 9.2
F7	230.1 ± 28.4	0.3 ± 0.03	−36.4 ± 17.3
F8	270.9 ± 16.3	0.5 ± 0.1	−35.2 ± 10.3

^a^Denotes the optimum formulation

Using Tween 80 (1% w/v) as a stabilizer, various solid: liquid lipid ratios were tested using different ratios, including 90:10, 70:30, 60:40, and 50:50 w/w. F5–F8 represented different NLC formulas loaded with IC and prepared using the previously mentioned ratios. F6, which was prepared using a ratio of 90:10 (234.1 ± 11.4 nm), had a higher particle size compared to F5 (173.9 ± 8.9 nm), which was prepared using a ratio of 70:30 (*P* < 0.05). F7 and F8, which were prepared using 60:40 and 50:50 ratios, exhibited a particle size of 230.1 ± 28.4 and 270.9 ± 16.3 nm, respectively. Both formulations showed a significant increase in size compared to F5 (*P* < 0.05). The incorporation of IC within F5, which showed the smallest particle size, did not result in a significant increase in size compared to the blank one (F4, 160.2 ± 12.3) (*P* > 0.05). Moreover, all the prepared NLCs exhibited a zeta potential around −30 mV or higher, as illustrated in Table 3.

### Preparation and optimization of Br-coated NLC

Four different concentrations of Br solution (0.2%, 0.5%, 0.7% and 2% w/v) in phosphate buffer (pH 4.5 or 6) were tested with three different ratios: 0.5:10, 1:10, 1.5:10, and 2:10 v/v Br solution to IC-NLCs, respectively. S1–S3 were prepared using 0.2% w/v Br solution (pH 4.5) using 0.5:10, 1:10, and 2:10 volume ratios, respectively. As shown in Table 4, a significant decrease in the negative zeta potential of the optimum uncoated formulation (F5, −30.2 ± 1.2) was observed, where S3 reached −13.1 ± 4.5 mV (*P* < 0.05). S4–S6 were prepared using 0.5% w/v Br solution (pH 4.5) using previously mentioned volume ratios. The negative ZP decreased in the case of the highest volume ratio (S6, 2:10) to reach −9.4 ± 3.3 mV, and a slight insignificant increase in size to reach 179.8 ± 15.1 nm and PDI value of 0.2 ± 0.1 compared to F5. This indicated the successful coating of NLC with Br solution as confirmed by a change in ZP without deteriorating the colloidal properties of nano-formulation. S7–S9 were prepared using a Br concentration of 0.7% maintaining the previously mentioned conditions. S9, which was prepared using a volume ratio of 2:10, did not exhibit any further increase in zeta potential (−9.1 ± 1.5 mV) compared to S6 (*P* > 0.05). However, the increase in the concentration of Br solution (from 0.5 to 0.7%) resulted in an unacceptable significant increase in particle size of S9 to reach 254.6 ± 10.3 nm and a PDI value of 0.5 ± 0.02 compared to S6 (*P* < 0.05). Therefore, S6 was chosen as the optimum formulation that was prepared using 0.5% w/v Br solution (pH 4.5) using a volume ratio of 2:10.

**Table 4:** Colloidal properties of Br-coated NLC prepared using different Br concentrations at different pH values and various volume ratios

Formulation code	Zeta potential	PS	PDI
S1	−18.7 ± 5.2		
S2	−11.4 ± 2.1		
S3	−13.1 ± 4.5		
S4	−14.4 ± 3.2		
S5	−13.7 ± 5.5	176.7 ± 12.2	0.2 ± 0.03
S6*	−9.4 ± 3.3	179.8 ± 15.1	0.2 ± 0.1
S7	−17.4 ± 7.2		
S8	−11.4 ± 2.5	298.9 ± 15.2	0.6 ± 0.3
S9	−9.1 ± 1.5	254.6 ± 10.3	0.5 ± 0.02
S10	−24.3 ± 12.2		
S11	−22.1 ± 7.5		
S12	−20.3 ± 6.6		
S13	−24.9 ± 3.7		
S14	−19.4 ± 2.5		
S15	−17.2 ± 3.3		
S16	−10.9 ± 4.2	350.2 ± 25.1	0.3 ± 0.1
S17	−6.6 ± 2.2		
S18	−5.5 ± 1.8	1171.2 ± 30.2	0.4 ± 0.1

*Denotes optimum formulation

In another set of experiments, the Br solution was prepared in phosphate buffer pH 6 using different concentrations and applied at different volume ratios. S10-S12 were prepared using 0.2% w/v Br using 0.5:10, 1:10, and 2:10 volume ratios, respectively. As shown in Table 4, a slight, insignificant increase occurred in ZP regarding the three formulations compared to F5 (*P* > 0.05), where the ZP of S12 that was prepared using the highest volume ratio was −20.3 ± 6.6 mV. Increasing the concentration of Br solution from 0.2–0.5% did not result in a significant increase in zeta potential (S15, zeta potential −17.2 ± 3.3 mV) compared to S6 (*P* > 0.05). S16-S18 were prepared using a higher concentration of Br solution (2%) using 1:10, 1.5:10, and 2:10 volume ratios, respectively. The three formulations showed a significant increase in zeta potential, where S18 reached −5.5 ± 1.8 mV, but with an unacceptable increase in size. S16 reached 350.2 ± 25.1 nm, and S18 was out of the nano-range. Accordingly, S6 was chosen as the optimum formulation.

### In vitro characterization of NLCs

#### Determination of Entrapment efficiency (EE%) of IC-NLC

The EE% of the selected optimum NLC (F5) was measured using an ultrafiltration technique. The IC-NLC displayed an EE% of 92.6%. The relatively high entrapment efficiency was maintained in the presence of the Br coat, as EE was 91%.

#### Microscopical examination of IC-NLC and Br-coated NLC

**Fig. 1:**
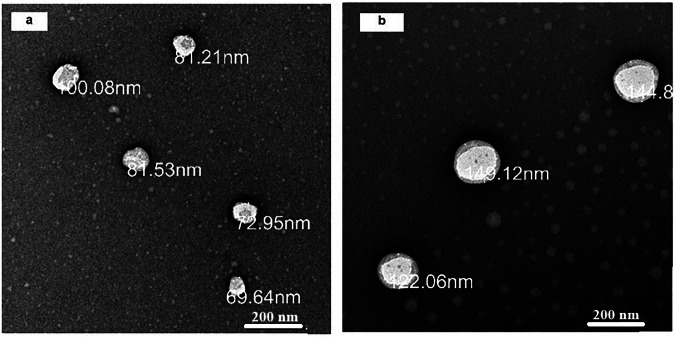
Morphological examination of **a** IC-NLC and **b** Br-NLC loaded with IC using transmission electron microscope (TEM) at a magnification power (×30 magnification power)

TEM imaging of the selected uncoated (F5) and Br-coated IC-NLC (S6) formulations presented nearly spherical and homogenously distributed nanostructures without aggregation, as demonstrated in Fig. 1. The microscopical examination of F5 revealed a particle size ranging from 70–100 nm, where Br-coated NLC showed an increase in particle size to reach 150 nm, as revealed in Fig. 1b. Additionally, no IC crystals were found during TEM examination of both formulations, further confirming efficient drug entrapment in the prepared NLC formulations.

#### Br coating efficiency

The concentration of free uncoated Br was detected in the filtrate using Bradford reagent. This reagent is used to measure total protein concentration using a spectrophotometric method [32]. The concentration of free Br in the filtrate was about 0.274 mg/mL; thus, the coating efficiency was about 73%.

#### Fourier transform infrared spectroscopy (FTIR) spectrum

**Fig. 2:**
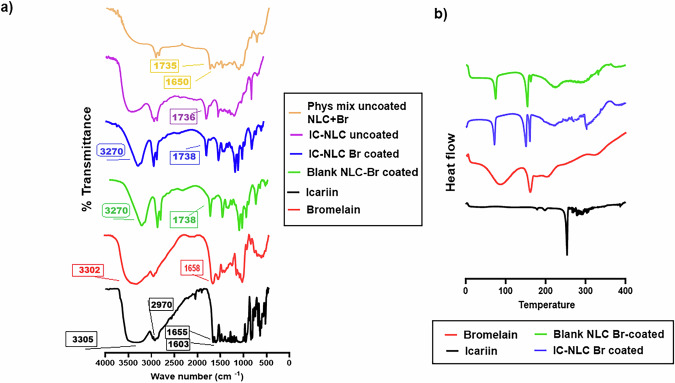
**a** FTIR spectrum of crude IC, Br, lyophilized uncoated IC-NLC, physical mixture of Br with lyophilized IC-NLC, Br-coated blank NLC, and Br-coated IC-NLC. **b** DSC chart of crude IC, Br, Br-coated blank NLC, and Br-coated IC-NLC

As illustrated in Fig. 2a, the FTIR spectrum of pure IC showed characteristic peaks at 3305, 2970, 2865, 1655, 1603, 1348, 1259, and 1185 cm^−1^. The 3305 cm^−1^ broad band indicated the stretching vibration peak of the phenolic hydroxyl group in IC. The spectrum 1650 cm^−1^ illustrated a C=C stretching vibration peak in IC, 1600 cm^−1^ was a C=O stretching vibration peak, and a strong peak was formed by the conjugation effect of C=C and C=O; 1260 cm^−1^ was a C–O extension vibration peak [26]. The spectrum of pure Br showed a characteristic peak at 3302 cm^−1,^ illustrating OH and NH overlapping bands. Another characteristic peak appeared at 1658 cm^−1^, indicating a strong intensity of C=O (amide I area) and a peak at 1547 cm^−1^ indicating C-N stretch band [33]. As illustrated in FTIR spectra, a peak appeared at 1735 cm^−1^ in blank/IC-loaded Br-coated and uncoated formulations, indicating the ester group of triglyceride in Compritol lipid “glyceryl behenate”. The physical mixture formed of Br with lyophilized uncoated formulation showed the significant ester peak of lipid at 1735 cm^−1^ and the significant amide band of Br at 1650 cm^−1^. The Br-coated Blank NLC showed a significant peak at 3270 cm^−1^ that may indicate OH or NH of Br. However, Br-coated blank and IC-loaded NLC did not show a significant amide band (no peaks appeared at 1650 cm^−1^). It is worth mentioning that the spectra of IC-NLC uncoated, IC-NLC-Br coated, and Blank NLC-Br coated share similar peak patterns, particularly in the fingerprint region (below 1500 cm^−1^) and the functional group region (around 3000–3500 cm^−1^). This suggests that the major chemical constituents (e.g., lipid matrix, stabilizers) are common across all samples, indicating a largely consistent formulation base. However, they are not similar. IC-NLC-Br-coated spectrum shows slight variations compared to the uncoated IC-NLC and blank NLC-Br, particularly around 1000–1300 cm^−1^, which may correspond to C–O–C stretches from the coating material. On the other hand, blank NLC-Br coated has slightly more intense or broader peaks in the fingerprint region, suggesting the presence of the coating without the drug.

#### Differential scanning colorimetry (DSC)

The obtained DSC spectrograms (Fig. 2b) illustrated the highly crystalline nature of IC as confirmed by the presence of a sharp endothermic peak at 249.59 °C (Δ*H* = 51.2036 J/g) [34]. The sharp melting endothermic peak, whose onset was at 147.4 °C, and the sharp peak at 155.4 °C (Δ*H* = 20.9 J/g) characterized the thermal behavior of Br [35]. The spectrogram of Br-coated blank/IC-NLC exhibited the presence of a significant Br peak and disappearance of the IC peak.

#### X-ray diffraction (XRD) analysis

**Fig. 3:**
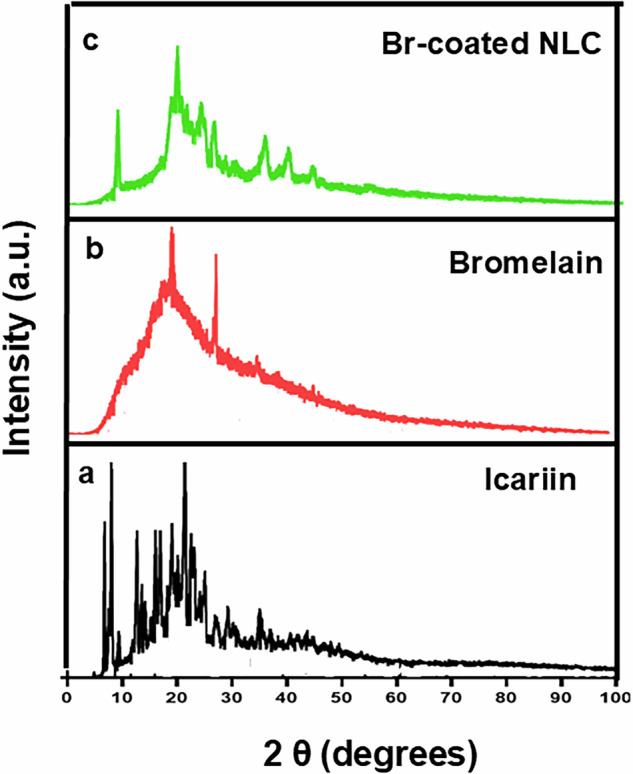
XRD chart of **a** crude IC, **b** Br, and **c** Br-coated IC-NLC

The obtained XRD pattern of crude IC confirmed its crystalline nature as illustrated in Fig. 3a. The XRD pattern showed the characteristic peaks of IC at 2θ 4.9, 7.9, 8.03, and 12.7°. The presence of these significant peaks was also reported by Lina Jia et al. [36]. Moreover, more significant peaks appeared at 2θ 21.4 and 22.6, similar to the reported peaks in patent number CN116410168A [37]. The XRD pattern of Br illustrated some low intensity peaks at 2θ 12.5, 16.2, and 23.8° [35] as shown in Fig. 3b. The XRD chart of Br-coated IC-NLC (Fig. 3c) illustrated the significant pattern of Br. Most of the prominent peaks that are characteristic of IC have diminished. The peak that appeared at 2θ 21.2° and a smaller peak at 2θ 23.4° may indicate the presence of Compritol.

#### In vitro release pattern of IC-NLC and Br-coated NLC loaded with IC

**Fig. 4:**
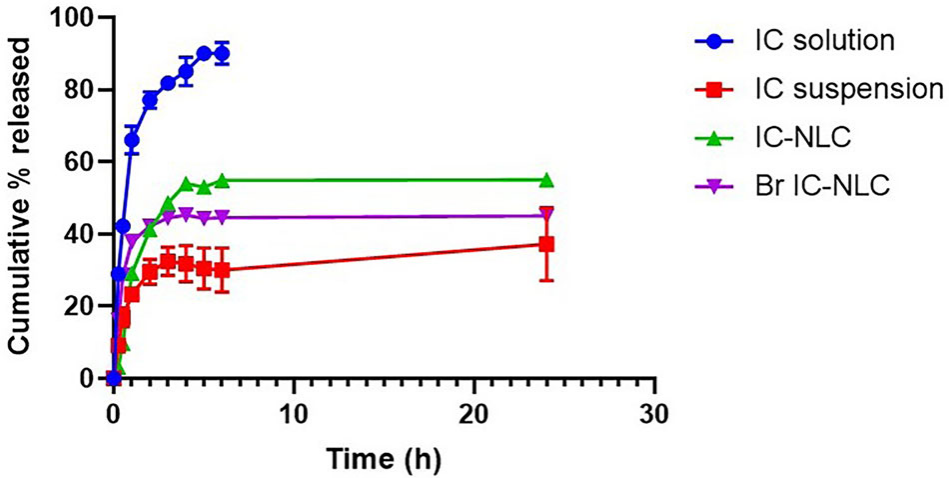
Release profile of IC solution, suspension, IC-NLC, and Br-coated IC-NLC nano-formulations in 30% v/v methanol in PBS

The solubility study illustrated the poor solubility of IC in PBS (2.3 µg/mL). The addition of 0.5% and 1% w/v Tween 80 enhanced solubility to reach 205.2 ± 1.1 µg/mL and 300.3 ± 2.2 µg/mL, respectively. Moreover, the solubility of 30% v/v methanol and 15% v/v methanol in PBS was 346.1 ± 3.1 µg/mL and 25.5 ± 0.4 µg/mL, respectively. Thus, 30% methanol in PBS release medium fulfilled the highest solubility and was the selected medium. As illustrated in Fig. 4, ~80% of the IC solution was released after only 2 h. A biphasic release pattern was observed with NLCs. A burst release was detected after 2 h in both coated and uncoated NLC, where 42.2 and 41.2% of IC was released, respectively, without a significant difference between both.

The mechanism of IC release from the uncoated and Br-coated NLC was analyzed using the Excel add-in DD solver by fitting to different release kinetics models. Results showed that the release data fit well to the Weibull model kinetic equation (*R*^2^ = 0.9, AIC = 52 and *R*^2^ = 0.9, AIC = 43 for uncoated and Br-coated NLC, respectively) as shown in Table 5.

**Table 5:** Release kinetic models illustrating *R*^2^ and AIC of each model

		Uncoated NLC	Br-coated NLC
First order	*R*^2^	0.3	0.7
	AIC	72	77
Zero order	*R*^2^	0.5	0.4
	AIC	83	83
Higuchi	*R*^2^	0.3	0.6
	AIC	72	73
Korsmeyer-Peppas	*R*^2^	0.7	0.6
	AIC	66	55
Weibull^a^	*R*^2^	0.9	0.9
	AIC	52	43

^a^Denotes best fit kinetics model

#### Stability study

The optimum formulations IC-NLC (*F4*) and Br-coated IC-NLC (*S6*) were stored at 4 °C for 3 months to detect any changes in their colloidal properties upon storage. As shown in Table 6, a slight, non-significant increase in size and PDI of uncoated NLC occurred upon storage (*P* > 0.05). The uncoated NLC showed a negative ZP (ranging from −27 to −30) throughout 3 months. Concerning Br-coated NLC, a slight increase in negativity occurred in the ZP of these NLCs upon storage. Moreover, a non-significant increase in size was observed by the end of the 3-month storage period (*P* > 0.05).

**Table 6:** Colloidal properties of coated and uncoated NLC upon storage at 4 °C for 3 months

Time	Colloidal properties of NLC			Colloidal properties of Br-coated NLC		
	Size (nm)	PDI	Zeta potential (mV)	Size (nm)	PDI	Zeta potential (mV)
0	173.9 ± 9.1	0.2 ± 0.1	−30.2 ± 1.2	179.8 ± 15.3	0.2 ± 0.1	−9.4 ± 3.3
1 month	185.3 ± 10.5	0.3 ± 0.2	−28.2 ± 3.4	215.2 ± 20.1	0.4 ± 0.3	−10.5 ± 9.9
3 months	190.2 ± 17.7	0.4 ± 0.3	−27.9 ± 7.6	220.4 ± 18.3	0.4 ± 0.5	−12.2 ± 15.4

### Cell culture

#### Cell viability assay

The viability of DOX-treated cells decreased to 44–51%. The addition of 2.8 µM of free IC, IC-NLC, and Br-coated IC-NLC led to an increase in viability by an increment of 10, 18, and 29% respectively. By applying a concentration of 11.25 µM, blank NLC and Br-coated blank NLC resulted in an increase in viability of 13 and 19% respectively. There was no significant difference between the effect of blank formulations and free IC solution (*P* > 0.05).

**Fig. 5:**
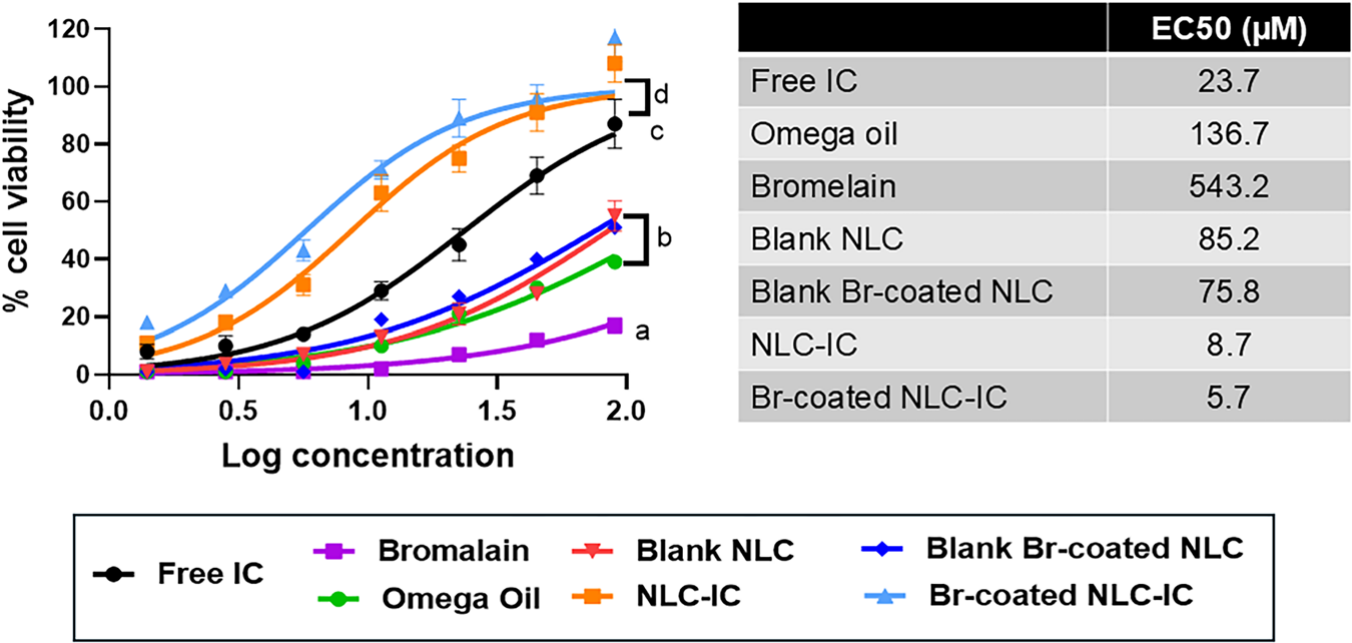
The effect of free IC and different formulations on the viability of DOX-treated cells using different concentrations and their calculated EC50. Results were significantly different when compared to the Free IC solution group at 11.25 µM, where *p* ≤ 0.05 at mean values: *a* < *b* < *c* < *d*

The IC concentrations that led to 50% (EC_50_) increase in cell viability were 23.7, 8.7, and 5.7 µM for IC solution, IC-NLC, and Br-coated IC-NLC, respectively. The Br-coated IC-NLC exhibited the lowest EC_50_ followed by IC-NLC, and both formulations were more potent than the free drug. The EC_50_ of blank NLC, Br-coated blank NLC, omega-3, and Br solution were 85.2, 75.8, 136.7, and 543.2 µM, respectively. These results elaborated the adequate bioactivity of omega-3 (EC_50_ 136.7 µM), which was better than that of the Br solution (EC_50_ 543.2 µM), as evident by the values of EC_50_. The Br-coated blank NLC achieved the lowest EC_50_ (75.8 µM) compared to uncoated blank formulations, omega-3, and Br solution, which illustrated the superior synergistic effects of combining both Br and omega-3 within NLC. The results were illustrated in Fig. 5 using serial dilutions of formulations.

Moreover, results illustrated in Table 7 demonstrated the synergistic effect of IC and omega-3 when applied together in the form of uncoated IC-NLC, where the CI = 0.6, indicating synergy. Moreover, the Br-coated IC-NLC exhibited a lower CI of 0.5. In addition, the application of uncoated IC-NLC resulted in a dose reduction in IC and omega-3, as clarified by the DRI value, which was 1.83 and 14.54, respectively. Moreover, a further dose reduction was observed in Br-coated IC-NLC, where DRI values for IC, omega-3, and Br were 2.5,19.9, and 131.8, respectively.

**Table 7:** The combination index and dose reduction indices calculated using CompuSyn software for both uncoated and coated IC-NLC

	CI	DRI		
		IC	Omega-3	Br
Uncoated IC-NLC	0.6	1.8	14.5	–
Br-coated IC-NLC	0.5	2.5	19.9	131.8

When CI < 1, this indicates synergy

#### Cellular uptake

**Fig. 6:**
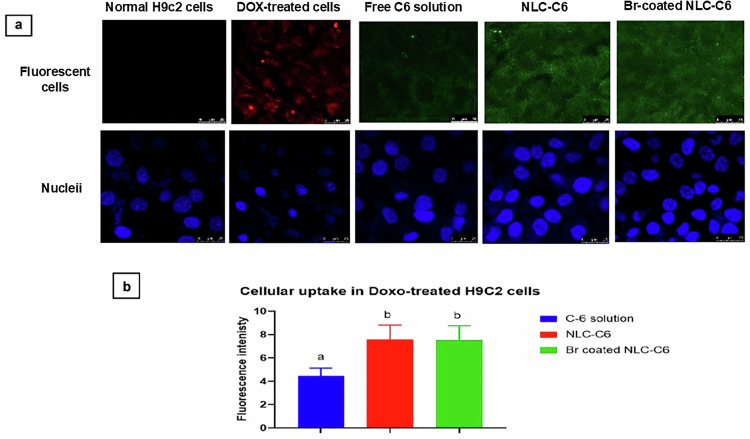
**a** Confocal microscopy showing red fluorescence of DOX and green fluorescence of C6 solution, C6-NLC, and Br-coated C6-NLC upon incubation of DOX-treated H9C2 cells for 4 h to evaluate cellular uptake. **b** Quantification of C6 fluorescence intensity by Image J. Results were significantly different where *p* ≤ 0.05 at mean values: *a* < *b*

The cells were treated with DOX, followed by the addition of C6 solution and formulations, where incubation lasted for 4 h. The fluorescence intensity of C6-NLC and Br-coated C6-NLC was significantly higher than that of C6 solution (*P* < 0.05), as illustrated in Fig. 6. It was notable that the Br coating did not affect the cellular uptake, as there was no significant difference in fluorescence intensity between coated and uncoated nano-formulations.

#### Apoptosis assay

**Fig. 7:**
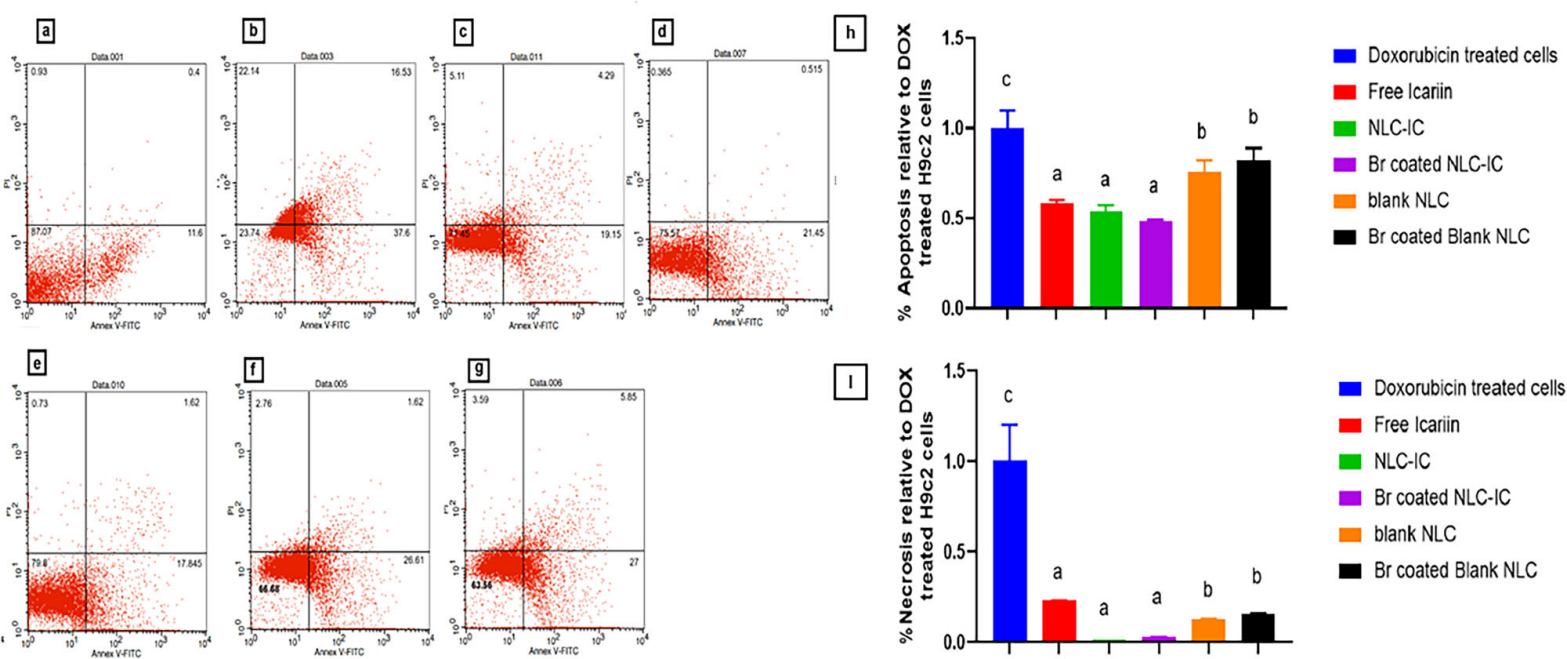
Cytofluorimetric evaluation of the effect of IC against Dox-induced apoptotic cell death. **a** control untreated cells, **b** DOX-treated cells, **c** cells pretreated by free IC, **d** cells pretreated by IC-NLC, **e** Br-coated IC-NLC, **f** blank NLC, and **g** Br-coated blank NLC. **h** The % total apoptosis rate of cells treated by different formulations relative to untreated H9c2 cells, and **i** the necrotic rate as % relative to control untreated cells. Results were significantly different where *p* ≤ 0.05 at mean values: *a* < *b* < *c*

As shown in Fig. 7a, b, DOX managed to significantly reduce viable cells to 37.6%, while the percentage of viable cells in the case of normal H9C2 was 87.1%. Additionally, DOX increased the total apoptotic rate (detected by early “quadrant III” and late “quadrant IV”) by 3.4-fold compared to normal untreated H9c2 cells (P < 0.05). Moreover, the necrotic rate of DOX-treated cells was also significantly increased 23.8-fold compared to normal untreated cells (*P* < 0.05). As illustrated in Fig. 7c–e, the pretreatment of cells with free IC, IC-NLC, and Br-coated IC-NLC decreased the total % apoptosis relative to DOX-treated cells by 1.7, 1.8, and 2.1 folds, respectively. Thus, the Br-coated IC-NLC fulfilled the highest decrease in apoptotic rate, followed by IC-NLC. In addition, the blank NLC and Br-coated NLC managed to decrease total apoptotic rate by 1.3 and 1.22 folds compared to DOX-treated H9c2 cells (*P* < 0.05), as shown in Fig. 7f, g. Moreover, the necrotic rate dramatically increased upon the addition of DOX. IC-NLC and Br-coated IC-NLC decreased the necrotic rate of cells by 60.6 and 30.3 folds, respectively, compared to DOX-treated H9c2 cells (*P* < 0.05). The effect of nano-formulations on necrosis was more prominent compared to IC solution, which managed to decrease necrotic rate by 4.3 folds only compared to DOX-treated cells (*P* < 0.05) as demonstrated in Fig. 7i.

## Discussion

NLCs were successfully prepared as an ideal delivery platform for encapsulating IC, enabling passive targeting to the infarcted myocardium.

In this study, hot high-shear homogenization was the selected technique to prepare blank and IC-loaded NLCs. The hot high-shear homogenization technique has various advantages, including short production time, ease of production, organic solvent-free operation, and scale-up feasibility. Two cycles of homogenization, each lasting 5 min, were sufficient to yield NLCs within the nano-range. This was preferable as a higher number of cycles can cause a rise in the sample’s temperature, resulting in particle coalescence due to the high kinetic energy of the particles [38]. The total amount of lipid phase (fat and oil) was kept constant (3% w/v) with respect to the 10 mL formulation. Compritol 888 ATO was the selected solid lipid, and omega-3 was the chosen oil. Both solid and liquid lipid phases were mixed and heated above the melting point of the lipid mixture, which was around 55 °C (70:30 Compritol: omega-3), where the drug was added to the lipid phase. It was reported that the degradation of polyunsaturated fatty acids within omega-3 is insignificant at temperatures below 80–100 °C when applied for a short time [39]. Moreover, Shahparast et al. reported that heating of omega-3 fish oil during the preparation of emulsions did not lead to a fast increase in lipid oxidation reactions [40].

It was reported that Compritol 888 ATO had been used in various NLC formulations and exhibited different particle sizes ranging from 129 to 323 nm [41]. Omega-3 was selected as the bioactive liquid lipid in the formulation of NLC due to its potential cardioprotective effect through several mechanisms of action. Omega-3 is rich in polyunsaturated fatty acids as DHA. These polyunsaturated fatty acids have minimal side effects and are listed as “Generally Recognized as Safe” according to the Food and Drug Administration. Omega-3 essential fatty acids can decrease ischemia-induced myocardial damage [42]. They exhibit antithrombotic effects, lower triglyceride levels, and regulate gene expression while enhancing membrane fluidity [43, 44]. Their antioxidant properties protect cardiomyocytes from ROS-induced ischemia-reperfusion injury, while nitric oxide stimulation supports vasodilation and anti-atherosclerotic actions. Additionally, omega-3 interferes with inflammatory pathways by inhibiting interleukin synthesis and NF-kB signaling, reducing proinflammatory eicosanoids. [45]. Specialized pro-resolving mediators like resolvins further decrease inflammation and infarct size. Their incorporation into the phospholipid bilayer enhances membrane fluidity, reinforcing cardiovascular protection [46].

The antioxidant, anti-apoptotic, and anti-inflammatory effects of fish n-3 fatty acids in cardiovascular disorders were reported in several papers. It was proposed that omega-3 may prevent the development of HF. Meligy et al. reported the anti-fibrotic and anti-inflammatory effect of omega-3 in the cardiac tissue. In this study, the cardioprotective effect of mesenchymal stem cells and n-3 polyunsaturated fatty acids was compared in a gentamicin-induced cardiac degeneration rat model. They emphasized the potential effect of omega-3 in promoting differentiation of myocardial cells, decreasing oxidative stress, and cell apoptotic rate. However, the application of mesenchymal cells outweighed the use of polyunsaturated fatty acids [16]. Moreover, Uygur et al. also reported the protective effect of omega-3 against DOX-induced cardiotoxicity. This was also attributed to the antioxidant and anti-apoptotic effects of omega-3. In addition to the potential role of EPA and DHA in reducing triglyceride levels, they also play a role in decreasing the incidence of arrhythmia and the development of ischemic heart diseases. Thus, they generally exert cardiovascular beneficial effects [17].

Regarding the selection of an optimum surfactant, it was reported that an increase in the concentration of surfactant would result in a significant decrease in the size of the nanoparticle, as manifested by increasing concentration of poloxamer 407 during optimization (F1–F3). However, Tween 80 (F4) was preferred as it fulfilled the target size range using a lower concentration (1% w/v) compared to poloxamer 407 (1.5% w/v). It was reported that higher concentrations of surfactants can cause significant hypersensitivity and toxicity issues [47].

The choice of optimum solid: liquid lipid ratio was essential as this ratio can affect the colloidal properties of the nano-system. It normally varies between 70:30 and 99.9:0.1% w/w [47]. The use of a higher lipid to oil ratio (90:10, F6) yielded higher particle size compared to F5, which was prepared using a ratio (70:30). This was in accordance with the literature, as it was reported that a higher concentration of solid lipid could result in a higher particle size [48]. However, further decreases in the concentration of solid lipid (F7,60:40 and F8,50:50 ratios) did not exhibit any further reduction in size. Furthermore, they showed a significant increase in size range (P < 0.05) compared to that of F5 (70:30). This was also reported in literature, where some authors reported that there is no trend that could relate the solid/lipid ratio to the colloidal properties. Accordingly, the ratio of 70:30 was the selected ratio, and F5 was chosen as the optimum formulation fulfilling the required particle size and polydispersity index (PS; 173.9 ± 8.9 and PDI; 0.2 ± 0.1) in addition to being stable with no subsequent precipitation of IC. This ratio was in accordance with the literature that stated that the concentration of solid lipid should not exceed 80% to allow incorporation of enough oil to formulate NLC. In addition, the negative zeta potential of the optimum formulation (−30.2 ± 1.2) was sufficient to stabilize lipid nanoparticles through electrostatic repulsion. The application of different surfactants and variable solid/liquid lipid ratios did not result in a significant difference in zeta potential (*P* > 0.05). This moderate negative zeta potential, in addition to the steric stabilization of the utilized SAA, was sufficient to maintain the stability of the formulation. The limited loading capacity of IC within lipid nano-formulations was in accordance with the literature [49, 50]. Liu et al. [49] succeeded in loading 4.7 mg IC within lipid nanoparticles; however, by using around 600 mg lecithin, which was a relatively high amount.

Regarding the preparation of coated NLC, Br was selected as the bioactive coating material. Br is a crude extract derived from the pineapple plant (Ananas comosus L.) and is found in several parts of the plant, including its stem, fruit, leaves, and peel. However, it is known that stems produce large amounts of Br. Stem Br contains a mixture of proteolytic enzymes whose isoelectric point is around ~9.5. Its optimum pH ranges from 4–8, and its molecular weight is about 23.8–37 kDa.

Br has great therapeutic potential with fibrinolytic, proteolytic, antifungal, antibacterial, antithrombotic, and anti-inflammatory effects. In addition, it plays a role in the prevention and treatment of several cardiovascular diseases, including angina pectoris and myocardial infarction [18, 20]. Several studies have highlighted Br’s fibrinolytic properties, which contribute to the dissolution of atherosclerotic plaque. Its anti-inflammatory effects are linked to the reduction of interleukins, tumor necrosis factor (TNF), and prostaglandins, further supporting its role as a natural anti-inflammatory agent that promotes healing [51]. During myocardial ischemia, the Akt kinase and the members of the forkhead box transcription factor/protein (FOXO) transcription factor family regulate programmed cardiomyocyte death. Br has been reported to protect cardiac cells by activating the Akt-FOXO signaling pathway. This process involves increased Akt phosphorylation, facilitating its nuclear translocation, leading to subsequent phosphorylation of a FOXO member and ultimately inhibiting cell death signals. However, enzymes as Br are characterized by being unstable, where their activity can decrease over time. Therefore, immobilization can be an essential method to maintain their efficiency. Immobilization techniques for Br include its entrapment within hydrogels or nanoparticles, covalent immobilization, and adsorption onto chitosan matrix [52]. Therefore, modification of the surface of the developed IC-NLCs with Br was performed in order to immobilize Br and improve the cardioprotective potential of IC.

In this study, for the first time, Br was used to coat NLC depending on the electrostatic interaction force between cationic Br solution and the negative surface of optimum NLC (F5 with zeta potential approximately −30.2 ± 1.2), leading to deposition of a Br layer around NLC. As previously mentioned, the isoelectric point of stem Br is around ~9.5; therefore, it will acquire a positive charge at pH values < 9. The Br solution was prepared at both pH 4.5 and pH 6. Efficient coating of NLC was assessed using changes in PS and ZP [53]. A decrease in the negativity of the Br-coated NLC indicated successful coating [24]. A simple titration technique was adopted to prepare Br-IC-NLCs as reported by Hassan et al., who used the same method for the preparation of chitosan-coated NLCs [23]. Our study illustrated that S10-S12, which were prepared using Br solution (pH 6), did not achieve satisfying results attributed to the lower positive charge density of Br prepared in pH 6 compared to that prepared in pH 4.5. It was reported that the density of Br positive charge was higher at lower pH values [54]. Thus, S6 was selected as the optimum formulation that was prepared using 0.5% w/v Br solution (pH 4.5) using a volume ratio of 2:10.

The in vitro characterization showed the relatively high entrapment efficiency of the selected optimum NLC (F5). This high entrapment efficiency was attributed to the lipophilic nature of IC. In addition, as NLC incorporated a proportion of liquid lipid (omega-3 oil), this might cause structural imperfection and disturbance in the crystalline arrangement of solid lipids. This would promote drug entrapment and prevent its leakage [55].

Moreover, the Br coating efficiency was evaluated using a Vivaspin ultrafiltration method. These ultracentrifugal concentrators, whose MWCO was 100,000 Da, were selected to allow filtration of free uncoated Br, whose molecular weight is about 23.8–37 kDa [56]. This experiment proved the successful immobilization of Br on the surface of NLC. These results were in accordance with ZP results that also confirmed Br coating attributed to an increase in ZP.

The FTIR illustrated the significant bands of crude IC and Br. The physical mixture of lyophilized uncoated IC-NLC and Br showed the bands of Br and confirmed the presence of lipid via the band for the ester group in lipid triglyceride. Successful Br coating in blank and IC-loaded NLC was confirmed despite the diminished amide band. This may be attributed to the presence of lipid that can shield or weaken the amide band of the peptide backbone [57].

In addition, the DSC confirmed the successful Br surface modification attributed to the persistence of the Br significant peak in the coated blank/IC-loaded NLC. The sharp peak of IC was diminished in Br-coated IC-NLC, which confirmed the transition of crystalline IC into an amorphous state upon entrapment within NLC. Moreover, XRD confirmed the amorphous nature of Br owing to the absence of intense peaks [35]. The appearance of the Br significant pattern in the chart of Br-coated IC-NLC confirmed the successful surface modification. The disappearance of IC peaks in the chart of the formulation illustrates its transition into amorphous form upon encapsulation within NLC.

Based on the solubility study, 30% v/v methanol in PBS was selected as the release medium to ensure sink conditions.

In the conventional dialysis bag method, the drug initially separates from the nano-formulation and diffuses into the solution inside the dialysis bag, serving as the donor compartment. It then permeates through the dialysis membrane into the external release medium, or receiver compartment. Maintaining sink conditions is essential to facilitate efficient drug diffusion across the membrane, preventing it from becoming the rate-limiting step. This setup enables precise analysis of release kinetics, accurately reflecting the drug’s actual release from the nano-carrier [58]. The in vitro release profile of IC solution in 30% v/v methanol in PBS, IC suspension in water, IC-NLC, and Br-coated IC-NLC was evaluated in the selected release medium (30% v/v methanol in PBS). The release profile of the nano-formulations was compared to that of an IC solution prepared in 30% methanol in PBS, which facilitated rapid diffusion of the drug in its molecular form, ensuring system suitability. The illustrated burst release could be attributed to the drug being adsorbed on the surface of prepared NLCs or located near the surface rather than being entrapped in the lipid matrix. Moreover, the relative enhancement in release pattern of drug-loaded NLC could be ascribed to a less crystalline and less ordered lipid matrix owing to the presence of liquid lipid. The burst release was followed by a more sustained and controlled release manner as encapsulated IC within the semisolid core of NLC was retarded and slowly released [59]. The presence of Br caused a slight retardation in IC release compared to uncoated NLC. This slight retardation was due to the presence of the Br coat that represented an additional barrier and relatively increased diffusion path length for IC to be liberated in the release medium [60]. The release data fitted well with the Weibull release kinetics model, which is particularly useful for explaining time-dependent release behavior. The Weibull shape parameter (β) was 0.1 (<1), denoting that the release rate decreased over time, suggesting diffusion-controlled release. The Weibull model has been found to effectively describe drug release from NLCs [61].

The optimum formulations IC-NLC (*F4*) and Br-coated IC-NLC (*S6*) maintained their stability upon storage at 4 °C for 3 months. The uncoated NLC maintained its high negative ZP throughout 3 months, thus avoiding any particle aggregation and ensuring stability. The slight increase in size of Br-coated NLC was attributed to the low ZP that could lead to slight particle aggregation. Fortunately, the change in colloidal properties of both coated and uncoated NLCs was insignificant, ensuring their stability.

The cardioprotective effects of IC solution, blank/IC-NLC, Br-coated blank/IC-NLC, omega-3 oil, and Br solution upon DOX-treated H9c2 cells were evaluated. The Br-coated IC-NLC achieved the highest increase in viability owing to the use of two bioactive excipients besides the active phytomedicine “IC.” The enhancement in cell viability by applying 11.25 µM of blank NLC and Br-coated NLC proved the protective effect of bioactive excipients. It illustrated the potential effect of incorporating bioactive excipients within nano-formulation. Moreover, the effect of free omega-3 and Br solution was also examined, maintaining the same quantities used in the nano-formulations, where they also achieved an adequate enhancement in viability attributed to their bioactivity. However, the synergistic effect of IC and bioactive excipients being loaded within NLC fulfilled the best improvement in viability. The obtained results were in accordance with the literature. Scicchitano et al. reported the improvement in cell viability of DOX-treated H9c2 cells that were pretreated by IC using comparable concentrations (1,5, 10, and 20 µM) [27].

In addition, an analysis of the protective effect of IC, omega-3, and Br on the viability of DOX-treated H9c2 cells when applied separately or in combination as either uncoated or coated NLC loaded with IC was determined using CompuSyn software. A CI was calculated at the IC concentration that led to a 50% increase in cell viability “EC_50_,” where a CI value < 1, = 1, or > 1 indicates synergism, additive effect, or antagonism, respectively. Also, DRI was calculated, where values greater than 1 indicate effective dose reduction of drugs when applied in a mixture compared to their individual application. DRI indicates the number of reduction folds in the dose of drug or bioactive excipient when applied as a pure ingredient relative to their dose when applied as a component within NLC formulation. The lower value of CI achieved by Br-coated IC-NLC compared to IC-NLC emphasized the synergistic effect of combining omega-3 and Br as bioactive excipients in addition to IC. Moreover, the results indicated that the combination of IC, omega-3, and Br within NLC might decrease the dose of each ingredient required to accomplish the same protective effect on the viability of DOX-treated H9c2 cells as achieved by each component separately, using a higher concentration.

The cellular uptake study was performed using the green, fluorescent lipophilic dye, coumarin (C6) as a model drug. The cellular uptake of C6-loaded NLC, Br-coated C6-loaded NLC, and free C6 solution in DOX-treated H9c2 cells using a CLSM was assessed. The enhanced cellular uptake of NLC may be attributed to its low particle size and high surface area, which facilitates endocytosis of nano-formulation within cells [62]. It was reported that endocytosis plays a main role in the entry of nanocarriers within cells compared to free, unformulated drug that enters cells through diffusion [63, 64].

The effect of IC solution and IC-NLC pretreatment on the apoptosis rate of DOX-treated H9c2 cells was evaluated using flow cytometry. The prominent effect of pretreating DOX-treated H9c2 cells on the apoptotic rate was also reported by Scicchitano et al. The study examined the unformulated IC using a concentration of 1 and 5 µM. This study did not refer to the effect of IC on necrotic rates. The relatively better protective effect of nano-formulations compared to IC solution was attributed to the better cellular uptake. Despite the successful ability of IC-NLC in the reduction of apoptosis, Br-coated IC-NLC achieved the lowest apoptotic rate owing to the use of two bioactive excipients: omega-3 and Br. The prominent effect of bioactive excipients was also evident as the blank NLC and Br-coated NLC managed to decrease total apoptotic rate by 1.3 and 1.2 folds compared to DOX-treated H9c2 cells. Generally, the coated and uncoated NLCs achieved a satisfactory protective effect on DOX-treated H9c2 cells, emphasizing the effect of nanotechnology, the therapeutic effect of phytomedicine, and the application of bioactive excipients.

Thus, the formulation of NLC was carefully designed to enhance its therapeutic potential by strategically incorporating bioactive excipients alongside IC phytomedicine. This approach not only improved the poor physical properties of IC but also introduced omega-3 oil as a bioactive excipient—without relying on organic solvents—further reinforcing the biocompatibility of the system. The NLC was engineered to have a size of less than 200 nm, facilitating passive targeting of infarcted myocardium. Additionally, its surface was coated with Br, another bioactive excipient, to enhance its functionality. The hydrophilic nature of the coated NLC played a crucial role in prolonging circulation time, ensuring sustained therapeutic effects.

Fundamentally, the design philosophy centered around the integration of bioactive excipients to synergistically improve the formulation’s overall efficacy. Notably, this nano-formulation presents a cost-effective alternative to the complex and resource-intensive methods of cell-based therapies. However, given its novelty as a chemical entity, extensive preclinical studies are required to validate its pharmacological properties, safety profile, and therapeutic spectrum. Further research should focus on optimizing key parameters, such as nanoparticle concentration and administration route, using animal models. Long-term studies with thorough follow-ups are also essential to evaluate bioavailability, assess unintended tissue accumulation, and confirm efficacy before translating this innovative formulation into clinical.

## Conclusion and future perspectives

To the best of our knowledge, this is the first study to evaluate the cardioprotective effect of Br-coated NLC prepared using omega-3, and to elaborate on the potential role of bioactive excipients application. The NLC was successfully coated by Br using a simple titration method, depending on electrostatic interaction for the first time. The coated and uncoated NLC achieved promising results regarding enhancing the viability of DOX-treated cells and reducing the apoptotic/necrotic rate. This was fulfilled using minimal concentrations of phytomedicine IC. The synergistic effect of IC, omega-3 fatty acids, and Br, combined with the targeted delivery capabilities of NLCs, resulted in significant protection of cardiomyocytes against DOX-induced toxicity. Future studies can further explore the therapeutic potential of this nanoformulation by:

- Conducting in vivo studies in animal models of myocardial infarction to assess the efficacy of the NLCs in reducing cardiac damage and promoting cardiac repair.
- Investigating the underlying mechanisms of action, including the role of IC, omega-3 fatty acids, and Br in protecting cardiomyocytes.
- Translating this promising nanotechnology into clinical applications to improve the treatment of cardiovascular diseases.

By addressing these future directions, we can further advance the development of effective nanomedicine strategies for the prevention and treatment of cardiovascular diseases.
